# Unfavorable nutrient intakes in children up to school entry age: results from the nationwide German KiESEL study

**DOI:** 10.3389/fnut.2023.1302323

**Published:** 2024-01-23

**Authors:** Leonie Burgard, Sara Jansen, Clarissa Spiegler, Anna-Kristin Brettschneider, Andrea Straßburg, Ute Alexy, Stefan Storcksdieck genannt Bonsmann, Regina Ensenauer, Thorsten Heuer

**Affiliations:** ^1^Department of Nutritional Behaviour, Max Rubner-Institut (MRI) – Federal Research Institute of Nutrition and Food, Karlsruhe, Germany; ^2^Department of Child Nutrition, Max Rubner-Institut (MRI) – Federal Research Institute of Nutrition and Food, Karlsruhe, Germany; ^3^Department of Nutritional Epidemiology, Institute of Nutrition and Food Sciences, University of Bonn, Bonn, Germany

**Keywords:** energy intake, nutrient intake, toddlers, preschoolers, nutrition survey, Germany

## Abstract

**Background:**

Nutrition in the first years of life is a cornerstone for child development and long-term health, yet there is a lack of current data on energy and nutrient intake among toddlers and preschoolers in Germany.

**Objective:**

To analyze energy and nutrient intake in toddlers (1- to 2-year-olds) and preschoolers (3- to 5-year-olds) in Germany and compare the results with the Dietary Reference Values (DRVs) by the European Food Safety Authority.

**Design:**

Dietary intake was assessed by weighed food record data (3 + 1 day) of 890 children from the representative cross-sectional Children’s Nutrition Survey to Record Food Consumption (KiESEL), carried out in 2014–2017 as a module of the German Health Interview and Examination Survey for Children and Adolescents Wave 2. For the calculation of energy and nutrient intake, the German Nutrient Database BLS 3.02, LEBTAB, and a supplement database were used.

**Results:**

Median intakes of energy and most nutrients met or exceeded the DRVs in both toddlers and preschoolers. However, low intakes relative to DRVs were found for vitamin D (6–9% of DRV, including supplements) and iodine (57–65% of DRV). Age specific downward deviations were observed for iron intake in toddlers (75% of DRV) and for calcium intake in preschoolers (67–77% of DRV). In contrast, intakes were high for saturated fatty acids (SFA) (14–16 E%), mono-/disaccharides (60–87 g/day), and protein [2.1–2.6 g/(kg body weight*day)].

**Conclusion:**

Nutrient imbalances in toddlers and preschoolers in Germany, which are partly age-related, give rise to concern. Research is needed to determine if routine vitamin D supplementation should be extended beyond infancy. Public health efforts to increase the rate of use of iodized salt and to reduce the intake of SFA and mono-/disaccharides in children’s diets are to be strengthened.

## 1 Introduction

Nutrition is a key factor in child development (1), substantially influencing not only physical but also mental and cognitive health (2). Moreover, early life nutrition has been found to have long-term effects on health, which include modulating the risk for non-communicable diseases such as obesity, diabetes mellitus, and cardiovascular disease (3, 4). At the same time, young children are particularly vulnerable to nutrient deficiencies, as nutrient requirements per kg body weight are high (5). In the midst of the overweight and obesity pandemic in the European Region, affecting an estimated 7.9% of children under the age of 5 and 29.5% of children aged 5 to 9 years (6), excessive energy and macronutrient intake appears to be accompanied with micronutrient deficiencies (7).

While the European Food Safety Authority (EFSA) has identified vitamin D, iron, and – in some countries – iodine as critical micronutrients among infants and young children below the age of 3 years (8), there is no such scientific opinion referring to critical nutrients in preschoolers. However, vitamin D and iodine are likely to be critical nutrients in older children as well, as an evaluation of the German *food-based* dietary guidelines for children and adolescents demonstrated that even adherence to the recommendations does not ensure adequate vitamin D and iodine intake (9).

Considering the long-term nature of nutrition-associated health consequences, the promotion of optimal nutrient intake in the earliest stages of life is pivotal. This in turn requires a comprehensive understanding of the various phases of child nutrition throughout early development and of potential levers for improvement. Yet, the last national dietary survey analyzing food consumption and nutrient intake in toddlers and preschoolers in Germany was the VELS study, carried out in children aged 1–4 years from 2001 to 2002 (10).

The Children’s Nutrition Survey to Record Food Consumption (*Kinder*-*Ernährungsstudie zur Erfassung des Lebensmittelverzehrs*, KiESEL), conducted between 2014 and 2017, offers the most recent representative data on food consumption for children aged 6 months to 5 years in Germany (11). Based on the KiESEL data, this study’s objective is to assess whether energy and nutrient intake in children aged 1–5 years in Germany comply with the Dietary Reference Values (DRVs) by EFSA (12). Furthermore, the study seeks to explore differences in nutrient intake specific to sex and age group, i.e., toddlers and preschoolers.

## 2 Materials and methods

KiESEL is a representative cross-sectional study performed by the German Federal Institute for Risk Assessment (*Bundesinstitut für Risikobewertung*, BfR) from 2014 to 2017. Originally, the study was designed to obtain current data on children’s food consumption for exposure assessment (11). Subsequent analysis of data on nutrient intake was performed by the Max Rubner-Institut (MRI). The study is a module of the German Health Interview and Examination Survey for Children and Adolescents Wave 2 (*Studie zur Gesundheit von Kindern und Jugendlichen in Deutschland Welle 2*, KiGGS Wave 2), which is part of the national health monitoring by the Robert Koch Institute (11). KiESEL was approved by the ethics committee of the Berlin Chamber of Physicians (Eth–28/13). Written informed consent was obtained from the primary caregiver of each child enrolled in the study. KiESEL was further approved by the German Federal Commissioner for Data Protection and Freedom of Information. To ensure adherence to the quality standards in nutritional epidemiology, the STROBE-nut reporting guidelines were used during manuscript preparation (13) (Supplementary Table 1).

The KiESEL sample was randomly selected from the gross sample of KiGGS Wave 2 (11). The sample of KiGGS Wave 2 was drawn from official residency registries of 167 representative German cities and municipalities originally chosen for the KiGGS baseline study (14). The total KiESEL sample includes *n* = 1104 children aged 0.5–5 years (11). The present analyses refer to a subsample of children aged ≥1 to ≤5 years (*n* = 890), after excluding children with missing food record data (*n* = 96) and infants aged ≥6 to ≤11 months (*n* = 118), as this age group is subject to a separate analysis. A participant flow chart is provided in Supplementary Figure 1. Children were assigned to two age groups based on their age at the beginning of data collection, namely toddlers (≥1 to ≤2 years) and preschoolers (≥3 to ≤5 years). Owing to the time lag between recruitment and data collection, the group of preschoolers additionally included *n* = 62 (6.2%) children aged 6 years. Note that all age specifications refer to completed years of life, e.g., the age group “1 year” refers to children aged 1.0–1.9 years. The KiESEL study design and survey protocol are reported elsewhere (11, 14).

Dietary assessment included a parent-administered food record, which was conducted on three consecutive days plus one independent day, scheduled 2–16 weeks later (3 + 1 design). To facilitate data collection, parents received face-to-face instructions during an initial home visit. They were provided with digital kitchen scales and a journal with pre-printed log pages explicitly inquiring about specific details of the foods and beverages consumed (e.g., preparation method, brand) and the place and time of the respective eating occasion. In cases where weighing was unfeasible, consumed amounts were estimated using package labels, household measures, or the KiESEL picture book visualizing different portion sizes. In child day-care facilities, a simplified food record was completed. If ambiguities were found in the protocol entries, the parents were contacted for clarification (11).

Data collection in KiESEL also included anthropometric measurements and a standardized questionnaire on nutritional behavior including a food propensity questionnaire, e.g., on seldomly eaten foods, which were performed by trained nutritionists during the home visit (11). To characterize the study population, data on socioeconomic status (SES) collected in KiGGS Wave 2 were used. The categories low, medium, and high SES reflect parental level of education, employment status, and income (equally weighted).

Amounts of human milk were estimated based on the age of the child and the frequency of feeding. Following the approach by Briefel et al. (15), the amount of human milk per feed was set at 89 ml for children aged 12–17 months and at 59 ml for children aged ≥18 months. The maximum daily human milk consumption observed in this KiESEL sub-sample was considered plausible, hence no upper daily limits were applied.

For the calculation of energy and nutrient intake, the food record data were either linked to the German Food Composition Database (*Bundeslebensmittelschlüssel*, BLS), version 3.02 (16), or to LEBTAB (17), considering all details of a food item as specified in the protocols (e.g., preparation method, brand). LEBTAB is a food composition database that contains a wide range of foods intended for infants and young children, such as follow-on formula or fortified toddler cereals. As far as included in the BLS, fortification of other foods, e.g., fruit juices and cereals, was also accounted for. Data on vitamin A are provided as retinol equivalents, vitamin E as α-tocopherol equivalents, vitamin K as phylloquinone, niacin as niacin equivalents, and folate/folic acid as folate equivalents.

The use of supplements was recorded using a free-text box within the food record (14). When the quantity and/or dosage of a supplement was not specified, amounts were derived as median from comprehensive protocol entries, referring to similar products in children of the same age. Protocol entries were linked to a supplement database (18), which was developed by the BfR and complemented by the MRI. To incorporate both dietary supplements and medicinal products such as vitamin D preparations for the prevention of rickets, the generic term “supplements” is used. The term “vitamin D-containing supplements” refers to all supplement preparations in which vitamin D has been specified as a component, i.e., mono and combination preparations with vitamin D. With the exception of vitamin D, nutrient intake from supplements was not considered in the analyses.

Following the EFSA protocol (19), misreporting of energy intake was identified using the Goldberg cut-off method updated by Black. Children aged ≥1 to ≤3 years were assigned a Physical Activity Level (PAL) of 1.4 and those ≥4 years a PAL of 1.6. Basal metabolic rate was calculated with the Schofield equations as a function of the child’s age, sex, height, and body weight (19). The ratio of reported energy intake to estimated basal metabolic rate was compared to calculated cut-off values (Supplementary Table 2). In line with the EFSA recommendation, under- and over-reporters were not excluded as this would introduce unknown bias (19).

A weighting factor was applied to approximate the sample’s sociodemographic structure to that of the German population. The weighting factor was developed by the Robert Koch Institute for the total KiESEL sample based on the factors sex, age, region, regional structure (e.g., rural area, large city), and household education level, fitted to data from official statistics (Microcensus 2015, except for household educational level Microcensus 2013 (20)).

For statistical analyses, the software SAS, version 9.4 (SAS Institute, Inc., Cary, NC, USA), was used. Statistical measures of energy and nutrient intake of the sample [median, 95% confidence interval (CI) of the median, and the 5th and 95th percentiles (hereafter P5 and P95, respectively)] were calculated from individual values derived as the mean of all protocol days per child. As nutrient intake distributions are frequently skewed, medians were calculated for each age group instead of means. Significant differences were identified by non-overlapping 95% CIs of medians for metric data and by chi-square tests (α = 0.05) for categorical data.

The DRVs by EFSA were used as measures for comparison (Supplementary Tables 3, 4). Intakes were additionally displayed as % of DRVs (median, interquartile range, minimum, maximum), derived from the individual intakes as % of sex- and age-specific DRV. In some cases, more than one DRV was applied for one KiESEL age group. For example, this was necessary for calcium, as the DRVs for calcium refer to 1- to 3-year-olds and to 4- to 10-year-olds, while the KiESEL age groups refer to 1- to 2-year-olds and to 3- to 5-year-olds.

Wherever possible, this report refers to the Population Reference Intake (PRI). In cases where PRIs have not yet been established, the Adequate Intake (AI) was used. Both are designed to cover the requirements of nearly all healthy individuals in a given reference population (12). Thus, an individual intake below a given reference value does not necessarily indicate an actual deficit but rather an increased probability of inadequate intake. Moreover, it should be noted that DRVs for young children are often derived from extrapolations from other age groups due to lacking data (21). DRVs for energy are provided as Average Requirements (ARs), whereas those for fat and carbohydrates are set as Reference Intake Ranges for Macronutrients (RIs) (12).

## 3 Results

### 3.1 Sample characteristics

The characteristics of the sample are described in Table 1. Compared to medium and high SES, the lowest proportion of children came from families with a low SES (<15%). Regarding misreporting of energy intake, 5.6% of parents were identified as under-reporters and 1.1% as over-reporters of their children’s food consumption. Supplement use was frequent only in 1-year-olds, with supplement use being reported for one in three at least once during the protocol period (Table 2). Among those, vitamin D-containing supplements were most commonly administered.

**TABLE 1:** Characteristics of KiESEL toddlers and preschoolers^1^.

	Toddlers (1–2 years; *n* = 354)	Preschoolers (3–5 years; *n* = 536)
**Sex (*n*, %)**		
Male	175 (51.6)	279 (51.4)
Female	179 (48.4)	257 (48.6)
**Anthropometric measurements (mean ± SD)**		
Body weight (kg)	12.2 ± 2.1	18.6 ± 3.6
Body height (cm)	85.7 ± 7.0	107.9 ± 8.8
BMI (kg/m^2^)	16.5 ± 1.6	15.8 ± 1.6
**Socioeconomic status (n, %)^2^**		
Low	19 (14.3)	34 (12.4)
Medium	205 (61.0)	330 (65.7)
High	130 (24.6)	169 (21.9)
**Region (n, %)^3^**		
North	50 (16.1)	61 (16.2)
East	129 (19.2)	171 (19.2)
South	93 (29.3)	164 (29.2)
West	82 (35.4)	140 (35.4)

^1^Weighted data (*n* unweighted). The age group toddlers refers to children aged ≥1 to ≤2 years and the age group preschoolers refers to children aged ≥3 to ≤5 years, but also includes 62 children of 6 years of age.

^2^Data on SES were missing for *n* = 3 children.

^3^Federal states were assigned as follows. North: Schleswig-Holstein, Hamburg, Lower Saxony, Bremen; East: Berlin, Brandenburg, Mecklenburg-Western Pomerania, Saxony, Saxony-Anhalt, Thuringia; South: Baden-Wuerttemberg, Bavaria; West: North Rhine-Westphalia, Hessia, Rhineland-Palatinate, Saarland.

**TABLE 2:** Supplement use in KiESEL toddlers and preschoolers^1^.

Age	Total participants (*n* = 890)	Supplement use (*n*, %)	Vitamin D-containing supplement^2^ use (*n*, %)
Toddlers	354	74 (21.7)*	63 (19.5)*
1 year	190	58 (32.5)	54 (31.1)
2 years	164	16 (10.3)	9 (7.2)
Preschoolers	536	41 (5.0)*	30 (3.8)*
3 years	147	10 (7.3)	8 (5.8)
4 years	163	19 (3.9)	13 (2.3)
5 years^3^	226	12 (4.0)	9 (3.4)

^1^Weighted data (n unweighted). Note that all age specifications refer to completed years of life, e.g., the age group “1 year” refers to children aged 1.0–1.9 years.

^2^Referring to mono and combination preparations with vitamin D.

^3^Incl. A total of 62 children of 6 years of age.

^*^Significant difference between toddlers and preschoolers.

### 3.2 Energy and macronutrient intake

**FIGURE 1:**
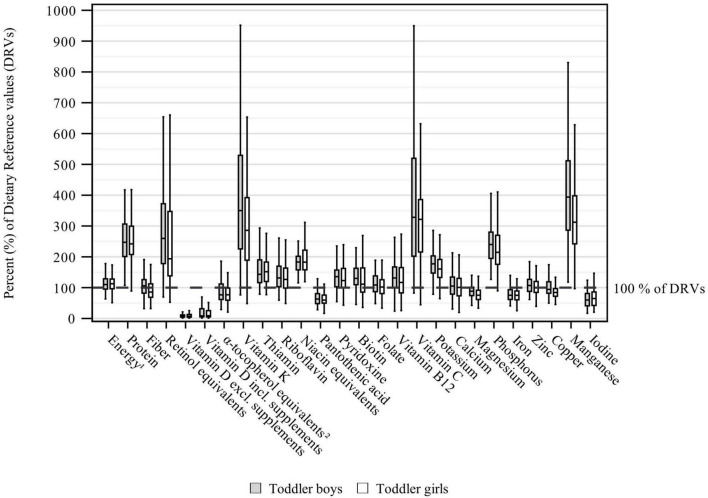
Daily energy and nutrient intake from food and beverages in toddlers (aged ≥1 to ≤2 years) stratified by sex and expressed as % of the EFSA DRVs (12) (weighted data; box and whisker plots with median, interquartile range, and minimum-maximum; whisker length limited to 1.5 times the interquartile range, outliers excluded). ^1^Assuming a PAL of 1.4.^2^The EFSA DRVs include **α**-tocopherol only, while KiESEL intakes are given as α-tocopherol equivalents.

**FIGURE 2:**
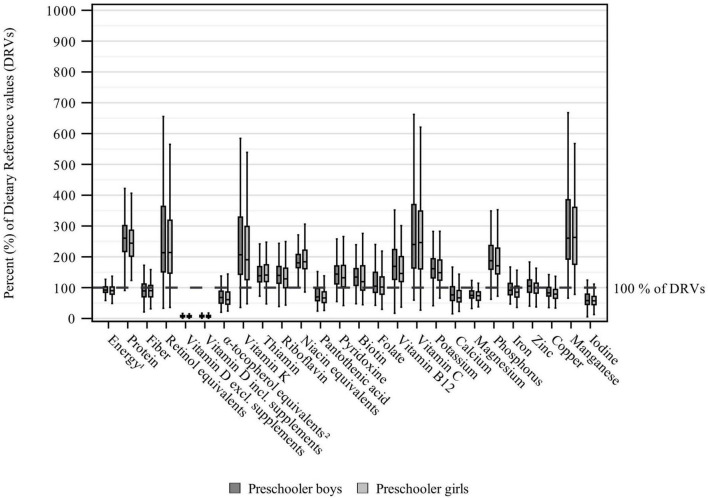
Daily energy and nutrient intake from food and beverages in preschoolers (aged ≥3 to ≤5 years) stratified by sex and expressed as % of the EFSA DRVs (12) (weighted data; box and whisker plots with median, interquartile range, and minimum-maximum; whisker length limited to 1.5 times the interquartile range, outliers excluded). The age group preschoolers refers to children aged ≥3 to ≤5 years, but also includes 62 children of 6 years of age. ^1^Assuming a PAL of 1.4 for preschoolers 3 years of age and a PAL of 1.6 for preschoolers ≥4 years of age. ^2^The EFSA DRVs include α-tocopherol only, while KiESEL intakes are given as α-tocopherol equivalents.

Daily energy and nutrient intakes in toddlers and preschoolers are depicted in Tables 3, 4. Median daily energy intakes were in the range of the respective sex- and age-specific ARs for both toddlers and preschoolers (Supplementary Table 3 and Table 3). With regard to intakes expressed as % of DRVs (Supplementary Table 4 and Figures 1, 2), protein intakes per kg body weight corresponded to about 2.5 times the PRIs in both toddlers and preschoolers, while carbohydrate intakes were within the RIs (Supplementary Table 4 and Table 3). Fat intakes were below the RI in toddlers but not in preschoolers. Also, median fiber intakes in toddler girls (but not in toddler boys) and preschoolers fell short of the DRVs and corresponded to 85 and 90% of the AIs, respectively. Mono-/disaccharides accounted for about half the total carbohydrate intake and made up approximately a quarter of the total energy intake (Table 3). Regarding fatty acids, SFA contributed to about 15 percent of energy intake (E%).

**TABLE 3:** Daily energy and macronutrient intake from food and beverages in KiESEL toddlers and preschoolers stratified by sex^1^.

	Toddlers (1–2 years; *n* = 354)						Preschoolers (3–5 years; *n* = 536)					
	**Boys (*n* = 175)**			**Girls (*n* = 179)**			**Boys (*n* = 279)**			**Girls (*n* = 257)**		
	**Median**	**CI Median**	**P5, P95**	**Median**	**CI Median**	**P5, P95**	**Median**	**CI Median**	**P5, P95**	**Median**	**CI Median**	**P5, P95**
Energy (kcal)*	979	937–1027^a^	689, 1410	916	868–922^a^	576, 1300	1297	1283–1334^a^	876, 1670	1188	1156–1215^a^	831, 1570
Protein (g)*	32.2	28.8–33.6	19.0, 45.1	30.0	28.4–32.8	16.1, 43.7	41.9	40.9–43.1^a^	26.5, 59.6	39.0	37.5–39.7^a^	26.3, 53.4
Protein (E%)	12.7	12.4–13.3	9.6, 16.4	13.0	12.2–13.3	9.5, 17.1	13.1	12.9–13.4	9.9, 15.8	13.0	12.7–13.3	10.4, 17.2
Protein (g/kg body weight)^2^*	2.6	2.4–2.7	1.5, 4.0	2.5	2.4–2.7	1.4, 3.9	2.3	2.2–2.4	1.6, 3.2	2.1	2.0–2.2	1.5, 3.5
Fat (g)*	36.1	33.3–38.0	22.5, 56.9	31.5	29.8–34.2	16.1, 50.2	47.7	45.7–49.8^a^	27.0, 73.0	42.7	39.6–44.2^a^	23.7, 65.4
Fat (E%)	32.7	32.0–33.7	24.8, 40.3	32.4	31.2–32.7	23.0, 41.2	33.3	32.4–34.1	23.6, 40.5	32.0	31.7–33.8	22.2, 42.0
Saturated fatty acids (g)*	17.3	16.1–18.2^a^	9.1, 27.9	15.1	13.3–15.8^a^	7.2, 26.6	22.1	21.2–23.1^a^	12.1, 34.3	19.8	18.9–20.9^a^	10.4, 31.2
Saturated fatty acids (E%)	15.9	14.7–16.7	10.3, 22.0	14.2	13.9–15.2	9.8, 20.8	15.5	14.6–16.0	9.8, 20.4	15.3	14.9–15.7	8.3, 20.8
Monounsaturated fatty acids (g)*	11.3	10.9–11.9^a^	6.8, 18.7	9.8	9.4–10.8^a^	4.5, 17.7	15.1	14.4–16.3	8.8, 24.0	13.8	13.4–14.5	7.0, 23.5
Monounsaturated fatty acids (E%)	10.3	10.1–10.8	7.9, 14.2	10.1	9.8–10.8	7.0, 15.3	10.7	10.5–11.1	7.5, 14.5	11.0	10.2–11.2	6.2, 15.9
Polyunsaturated fatty acids (g)*	4.1	3.9–4.5	2.5, 8.1	4.0	3.7–4.2	2.1, 9.1	6.1	5.8–6.3^a^	3.0, 11.1	5.1	4.9–5.6^a^	2.5, 10.2
Polyunsaturated fatty acids (E%)	4.1	3.9–4.4	2.4, 7.4	4.2	3.7–4.4	2.6, 7.0	4.1	3.9–4.3	2.5, 6.7	4.0	3.8–4.2	2.4, 7.1
Cholesterol (mg)*	128.8	120.2–138.9	42.9, 212.1	112.9	100.2–123.2	25.7, 205.8	169.3	162.8–176.7^a^	77.5, 299.9	145.8	138.8–159.1^a^	61.1, 332.2
Carbohydrates (g)*	127.6	125.1–132.0	91.5, 188.9	122.1	112.5–125.6	79.7, 177.3	170.5	166.1–177.0^a^	123.7, 229.5	156.8	150.9–162.9^a^	104.9, 214.2
Carbohydrates (E%)	53.1	52.5–53.4	44.4, 60.4	53.3	52.2–55.1	42.2, 64.3	52.5	51.5–53.9	44.3, 62.6	53.5	52.6–54.3	42.0, 63.3
Mono-/disaccharides (g)*	63.4	59.6–67.1	40.9, 108.8	59.9	54.4–62.2	27.5, 99.9	86.7	83.2–89.5^a^	44.4, 136.3	74.5	69.8–77.7^a^	43.5, 131.3
Mono-/disaccharides (E%)	25.3	24.4–27.2	17.4, 38.9	26.5	25.2–27.4	17.4, 38.4	27.1	26.4–28.6^a^	15.4, 40.1	24.4	24.2–26.4^a^	16.4, 38.0
Fiber (g)*girls only	10.6	10.1–11.1^a^	6.0, 17.2	8.6	8.2–9.3^a^	4.3, 16.9	11.3	10.8–12.0	6.9, 21.4	10.7	10.6–11.2	6.0, 17.8

^1^Weighted data (n unweighted). The age group toddlers refers to children aged ≥1 to ≤2 years and the age group preschoolers refers to children aged ≥3 to ≤5 years, but also includes 62 children of 6 years of age. Energy and nutrient intake was calculated using BLS 3.02 (for ordinary foods/beverages) and LEBTAB (for foods/beverages intended for infants/young children). CI Median, 95% confidence interval of the median; P, percentile. Due to the display of median values, the sum of protein, fat, and carbohydrate E% does not equal 100%.

^2^The EFSA DRVs are given in g per kg body weight.

^*^Significant difference between age groups (95% confidence intervals of the medians do not overlap).

^a^Significant difference between sexes (95% confidence intervals of the medians do not overlap).

**TABLE 4:** Daily micronutrient intake from food and beverages in KiESEL toddlers and preschoolers stratified by sex^1^.

		Toddlers (1–2 years; *n* = 354)						Preschoolers (3–5 years; *n* = 536)					
		**Boys (*n* = 175)**			**Girls (*n* = 179)**			**Boys (*n* = 279)**			**Girls (*n* = 257)**		
		**Median**	**CI Median**	**P5, P95**	**Median**	**CI Median**	**P5, P95**	**Median**	**CI Median**	**P5, P95**	**Median**	**CI Median**	**P5, P95**
Vitamins	Retinol equiv. (μg)	649	602–700	273, 1631	484	428–652	217, 1357	616	573–666	240, 2233	592	561–691	235, 1819
	Vit. D excl. suppl. (μg)*girls only	1.1	1.0–1.2	0.5, 6.0	1.1	1.0–1.3	0.3, 6.1	1.0	1.0–1.1^a^	0.4, 3.1	0.9	0.9–1.0^a^	0.3, 2.7
	Vit. D incl. suppl. (μg)*girls only	1.3	1.1–1.5	0.5, 14.8	1.3	1.1–1.7	0.3, 14.4	1.0	1.0–1.1	0.4, 5.4	0.9	0.9–1.0	0.3, 2.7
	α-TE equiv. (mg)*boys only	4.6	4.4–5.1	2.6, 11.2	4.6	4.3–5.2	2.1, 11.2	6.1	5.8–6.4	2.7, 14.9	5.5	5.2–6.0	2.8, 12.1
	Vit. K (μg)*boys only	42.0	37.6–45.9	16.1, 129.5	34.3	29.8–38.1	10.9, 119.0	34.3	31.9–36.4	15.9, 115.4	33.6	30.6–37.4	11.6, 105.8
	Thiamin (mg)*	0.60	0.56–0.64	0.35, 1.14	0.56	0.52–0.60	0.27, 1.22	0.74	0.73–0.79^a^	0.42, 1.74	0.69	0.67–0.72^a^	0.40, 1.83
	Thiamin (mg/MJ)^2^	0.14	0.14–0.15	0.09, 0.29	0.15	0.14–0.16	0.10, 0.31	0.14	0.13–0.15	0.09, 0.30	0.14	0.13–0.15	0.09, 0.35
	Riboflavin (mg)	0.79	0.74–0.88	0.46, 1.41	0.76	0.71–0.82	0.33, 1.51	0.91	0.87–0.98	0.50, 2.04	0.83	0.81–0.88	0.47, 1.70
	Niacin equiv. (mg)*	11.7	11.0–12.4	7.5, 19.8	11.6	10.7–12.9	6.7, 17.6	16.3	15.6–16.6	10.1, 28.7	15.0	13.8–15.9	9.6, 23.9
	Niacin equiv. (mg/MJ)^2^	2.93	2.77–3.02	2.20, 4.02	2.92	2.72–3.12	2.22, 4.23	2.89	2.82–2.93	2.23, 4.47	2.94	2.86–3.04	2.22, 4.65
	Pantothenic acid (mg)*	2.5	2.3–2.7	1.4, 5.2	2.4	2.2–2.5	1.0, 4.5	2.8	2.7–2.9	1.6, 6.6	2.7	2.6–2.7	1.4, 5.9
	Pyridoxine (mg)*	0.81	0.78–0.87^a^	0.48, 1.36	0.74	0.71–0.77^a^	0.33, 1.34	0.92	0.90–0.98	0.58, 2.17	0.89	0.87–0.96	0.50, 2.10
	Biotin (μg)*	26.0	24.3–27.8	16.0, 62.8	22.3	21.2–24.9	10.6, 64.4	31.4	30.2–33.3^a^	16.2, 119.1	29.3	27.8–30.1^a^	15.2, 119.9
	Folate equiv. (μg)	130	123–142	70, 227	122	116–128	57, 224	140	133–151	76, 328	141	127–149	71, 320
	Vit. B12 (μg)*	2.0	1.8–2.1	0.9, 3.6	1.8	1.5–2.0	0.6, 3.3	2.5	2.4–2.6^a^	1.1, 4.8	2.2	2.1–2.3^a^	1.0, 4.2
	Vit. C (mg)	65.7	58.7–73.7	23.2, 129.5	64.3	60.7–68.5	15.1, 128.4	66.5	63.8–71.7	21.6, 165.3	59.6	57.3–65.4	23.5, 160.7
Minerals	Sodium (g)*	1.00	0.93–1.09	0.40, 1.91	1.00	0.96–1.09	0.49, 1.88	1.44	1.42–1.50	0.81, 2.51	1.45	1.41–1.50	0.86, 2.21
	Potassium (mg)*	1418	1347–1494	913, 2139	1284	1239–1356	593, 2012	1597	1573–1624	964, 2410	1545	1511–1577	919, 2293
	Calcium (mg)	473	449–503	221, 793	454	391–518	210, 791	519	495–558	269, 952	485	467–519	276, 788
	Magnesium (mg)*	150	139–158^a^	87, 226	128	121–138^a^	75, 233	172	167–177	105, 265	170	158–178	110, 232
	Phosphorus (mg)*	600	541–626	390, 902	537	505–584	318, 849	741	716–757^a^	468, 1066	676	658–702^a^	436, 934
	Iron (mg)*	5.3	5.0–5.5	3.3, 9.1	5.3	5.0–5.5	2.4, 8.5	6.4	6.3–6.8	3.9, 11.7	6.0	5.9–6.3	3.4, 9.7
	Zinc (mg)*	4.6	4.5–4.9	3.1, 7.8	4.3	4.1–4.7	2.3, 6.4	5.4	5.2–5.6	3.5, 8.0	5.1	4.9–5.3	3.4, 7.3
	Copper (mg)*	0.66	0.64–0.73^a^	0.45, 1.04	0.59	0.55–0.62^a^	0.37, 1.16	0.83	0.81–0.88	0.52, 1.29	0.79	0.76–0.82	0.51, 1.18
	Manganese (mg)*girls only	2.0	1.8–2.1^a^	1.0, 3.3	1.6	1.5–1.7^a^	0.7, 3.9	2.2	2.0–2.3	1.2, 4.3	2.1	1.9–2.3	1.2, 3.6
	Iodine (μg)^3^	54.0	48.0–56.0	23.8, 111.8	58.2	51.4–63.0	19.4, 102.4	52.1	48.2–55.4	24.1, 120.2	51.7	50.9–55.0	28.6, 135.7

^1^Weighted data (n unweighted). The age group toddlers refers to children aged ≥1 to ≤2 years and the age group preschoolers refers to children aged ≥3 to ≤5 years, but also includes 62 children of 6 years of age. Nutrient intake was calculated using BLS 3.02 (for ordinary foods/beverages) and LEBTAB (for foods/beverages intended for infants/young children). CI Median, 95% confidence interval of the median; Equiv., equivalents; P, percentile; Vit., vitamin; α-TE, α-tocopherol.

^2^The EFSA DRVs are given in mg per MJ.

^3^Possibly underestimated, as iodized salt in family foods is not fully accounted for.

^*^Significant difference between age groups (95% confidence intervals of the medians do not overlap).

^a^Significant difference between sexes (95% confidence intervals of the medians do not overlap).

The difference in energy intake between boys and girls was more pronounced in preschoolers (median Δ 109 kcal) than in toddlers (median Δ 63 kcal). Consequently, sex-specific differences in daily median macronutrient intakes were predominantly observed in preschoolers (protein, fat, SFA, polyunsaturated fatty acids, cholesterol, carbohydrates, mono-/disaccharides) and less in toddlers (SFA, monounsaturated fatty acids, fiber). Differences in the contribution to energy intake (E%) were only found for mono-/disaccharides in preschoolers. For all differences, intake was consistently higher amongst boys than girls.

### 3.3 Micronutrient intake

Except for vitamin D, α-tocopherol equivalents, and pantothenic acid, median vitamin intakes met or exceeded the applicable DRVs in both toddlers and preschoolers (Figures 1, 2). The largest shortfall relative to the DRVs was found for vitamin D, with median intakes corresponding to less than 10% of the AI for both age groups and sexes, regardless of individual supplement use. Depending on age group and sex, median intakes of α-tocopherol equivalents corresponded to 61–77% and median intakes of pantothenic acid to 60–67% of the AIs, respectively. Total vitamin intakes were largely higher in preschoolers than in toddlers or showed no differences between age groups, except that girls’ vitamin D intakes and boys’ vitamin K intakes were higher in toddlers than in preschoolers. Sex-specific differences in vitamin intake were found more frequently in preschoolers (vitamin D without supplements, thiamin, biotin, vitamin B12) than in toddlers (pyridoxine), with consistently higher intakes in boys than in girls (Table 4).

Among the minerals, median intakes below the DRVs were found for iodine, iron, calcium (preschoolers only), magnesium, and copper. The largest gap in intake relative to the DRVs was found for iodine, with median intakes corresponding to 57–65% of the AI. Median iron intakes corresponded to around 75% of the PRI in toddlers. In preschoolers, median iron intakes were higher, at 92% of the PRI in boys and 85% of the PRI in girls. While median calcium intakes in toddlers met the DRV, the intakes in preschooler boys and girls corresponded to 77 and 67% of the PRIs, respectively, and did not meet the ARs either [390 mg/day for 3-year-olds and 680 mg/day for 4- to 6-year-olds (12)]. Relating to both age groups, median magnesium intakes were equivalent to 74–89% of the AI. For copper, median intakes reached 79–94% of the AI. Overall, mineral intake was higher in preschoolers than in toddlers. However, for calcium and manganese (in boys), the difference between age groups was not significant. Sex-specific differences were found for magnesium, copper and manganese in toddlers, and for phosphorus in preschoolers, all showing higher intakes in boys (Table 4).

## 4 Discussion

This representative study identified nutrient imbalances in young children in Germany up to school entry age, showing vitamin D and iodine intakes well below DRVs, irrespective of age and sex, as well as age-specific non-attainment of DRVs for iron in toddlers and calcium in preschoolers. In contrast, high intakes were found for SFA, mono-/disaccharides, and protein in both age groups.

For vitamin D, the majority of requirement is usually covered by endogenous synthesis in the skin. However, the EFSA AI is based on the premise of minimal cutaneous vitamin D synthesis (12) and may overestimate dietary requirements in case of sufficient sun exposure. According to the European Academy of Paediatrics (EAP), vitamin D deficiency is likely to affect a considerable proportion of healthy European children (22). For Germany, KiGGS data showed a prevalence of vitamin D deficiency (25-hydroxyvitamin D <30 nmol/L) of 5.7% (girls) and 4.9% (boys) in 1- to 2-year-olds and 9.1% (girls) and 11.5% (boys) in 3- to 6-year-olds (23). All European countries recommend vitamin D supplementation in infants (22). In some countries, this recommendation is extended to older children (24), but not in Germany (25). This is reflected by the higher proportion of vitamin D supplement users in KiESEL children of 1 year of age compared to children aged ≥2 years. Given the overall low percentage of supplement users in both age groups, vitamin D intake was likely inadequate with insufficient sun exposure. Vitamin D intakes reported for other European countries, seemed to be higher than in KiESEL, though still below the AI (26–34). In contrast to Germany, some of these countries have mandatory vitamin D fortification policies and/or a broader range of products to which vitamin D may be added voluntarily (35).

Iodine was confirmed as being another critical nutrient in both age groups. While iodine in fortified infant and toddler foods was considered, iodine from iodized salt could only be accounted for if explicitly reported for homemade dishes, as the preset recipes of the BLS contain non-iodized salt by default. However, according to an analysis of iodine exposure levels within the total diet BfR-MEAL-study, 1- to 6-year-olds in Germany have a high risk of inadequate intake, even under the premise of household use of iodized salt (36). A German regional cohort study (DONALD) found that the median 24-h urine iodine excretion in children aged 6–12 years decreased from 2012 onward and reached a minimum of 58.9 μg/d in 2018 (37), classified as mild iodine deficiency (38). This is thought to be due to a decrease in the use of iodized salt (37), described as a key iodine source in German preschoolers (39). Other European surveys showed iodine intakes twice as high (26, 27, 30, 31, 34), and in Danish preschoolers even three times as high as in KiESEL (32), likely explained by the mandatory iodine fortification of household salt and salt for commercial bread production in Denmark (40). In contrast, the use of fortified salt is voluntary in Germany (36). The German food-based dietary guidelines for children and adolescents recommend that households use iodized salt and choose foods with iodized salt over foods with unfortified salt for intakes to meet DRVs (9). In KiESEL, around 74% of parents stated using mainly iodized salt (36). However, the rate of use in the German food industry is estimated at 29% (41), which makes it challenging for households to choose foods with iodized salt. The apparently lower iodine intake in KiESEL in a European comparison may also be explained by mean consumption of milk and milk products (26, 30, 32, 34) and fish being lower (27, 30, 32, 34), which are important sources of iodine.

The present analysis also suggests age-specific deficits in intake for iron in toddlers and calcium in preschoolers. Though reaching only two-thirds of the PRI, toddlers’ iron intake seemed to be lower midfield in a European comparison (26–31) and met the AR of 5 mg/day (12). Worth noting, KiESEL infants (≥6 to ≤11 months) even had iron intakes less than the AR (own unpublished data, 2022). Thus, both infancy and toddlerhood appear to be associated with a higher likelihood of low iron intake than preschool age. One possible explanatory factor might be a higher consumption of meat and meat products at older ages [e.g., 5-year-olds showed an over 1.5 times higher meat consumption per kcal energy intake than 1-year-olds in KiESEL (own unpublished data, 2023)].

Calcium intake, on the other hand, appeared to be potentially critical in preschoolers only, which could be related to the PRI being considerably higher in preschoolers than in toddlers [800 vs. 450 mg/day (12)]. According to EFSA, a median intake equal to the AR reflects a risk of inadequate intake in 50% of individuals (42). Consequently, more than half of KiESEL preschoolers were at risk of insufficient calcium intake. Calcium intake in KiESEL preschoolers seemed lower than in other European surveys (27, 28, 32–34), which may too be related to mean consumption of milk and milk products being lower (28, 32, 34, 43).

In contrast, protein intakes in KiESEL toddlers and preschoolers might be too high. There is emerging evidence supporting a link between high protein intake in early life and later risk of obesity (44). However, in the absence of applicable upper intake levels for the period beyond complementary feeding, a final conclusion on protein intake is not possible. Though intakes in KiESEL exceeded DRVs, available data on protein intake (E%) from other European studies suggest them being at the lower end of the spectrum (26, 28–32, 34).

In the absence of an intake threshold below which no adverse effects exist, the EFSA recommends SFA intake to be as low as possible (12). The WHO set a recommended limit of 10 E% (45), which was clearly exceeded in both KiESEL toddlers and preschoolers, pointing to an unfavorable fatty acid pattern with regard to the risk of cardiovascular disease in later life (46). This observation is in line with a review concluding that the intake of SFA in children aged 1–7 years worldwide was mostly above recommended maximum thresholds, especially in Europe (47). In a European comparison, SFA intakes in KiESEL appeared to be mid-range (26, 28–30, 32, 34).

Similarly, the WHO recommends reducing free sugar intake to <10 E% (48), while EFSA could not identify a level of intake without adverse effects (49). A high intake is likely to facilitate adverse food preferences in early life, e.g., for sweet taste (5), and promote weight gain (48). In KIESEL, free sugars from soft drinks, sweets, fruit juices, cakes, milk and milk products, breakfast cereals, and spices/seasoning sauces corresponded to an estimated 12 E% in toddlers (in boys and girls) and 18 and 17 E% in preschooler boys and girls, respectively (own unpublished data, 2023), estimates derived as in Heuer (50). Intakes were thus too high, particularly in preschoolers. Based on an EFSA analysis, the mean free sugar intake in Europe ranged between 4 and 18 E% in toddlers and 8 and 20 E% in children aged 3–9 years (49).

The present analysis shows a number of differences in nutrient intake between boys and girls that are expected to be related to the higher energy intake in boys compared to girls. Worth noting is that on average preschool boys consumed disproportionately more mono-/disaccharides than their female peers. Boys in the older KiESEL age group consumed more sweets and soft drinks compared to girls (mean: + 19 g/day and + 42 g/day, respectively) (51), likely making them more affected by the adverse effects of free sugar intake.

With nutrition in the early years of life being a key determinant of lifelong health, it is fundamental from a public health perspective to rigorously invest in measures targeting this decisive early phase of life. The present study offers valuable guidance for public health service providers and policymakers as to which nutrients and groups at risk to prioritize and assists in ensuring efficient, need-based resource allocation.

Key strengths of this study are the representative sampling approach and the use of a weighting factor to correct for deviations from the German population, but also the level of detail of data provided by weighed food records (52). Besides, the joint use of the two food composition databases BLS and LEBTAB improves the matching of food items. However, despite the use of a weighting factor, children of parents with low SES were somewhat underrepresented, limiting generalizability. Also, weighed food records entail a high respondent burden, potentially inducing changes in dietary behavior (52), and may be confounded by social desirability bias (53). Besides, a comprehensive assessment of nutrient deficiency risk also requires the analysis of relevant biomarkers. However, feasibility is limited due to high costs, limited parental compliance (54), and a lack of reliable biomarkers (55).

## 5 Conclusion

Toddlers and preschoolers in Germany show nutrient imbalances consisting of non-attainment of several micronutrient DRVs (particularly vitamin D and iodine), accompanied by unfavorable macronutrient distribution (high share of SFA, mono-/disaccharides, and potentially also protein). Research is urgently needed to determine if routine vitamin D supplementation should be extended beyond infancy. Measures to increase the rate of use of iodized salt by both the food industry and households as well as to lower the intake of SFA and mono-/disaccharides during early childhood are to be strengthened.

## Data availability statement

The data analyzed in this study is subject to the following licenses/restrictions: Data described in the manuscript, code book, and analytic code will be made available upon request pending application and approval. Requests to access these datasets should be directed to TH, thorsten.heuer@mri.bund.de.

## Ethics statement

The study involving humans was approved by the Ethics Committee of the Berlin Chamber of Physicians (Eth-28/13). The study was conducted in accordance with the local legislation and institutional requirements. Written informed consent for participation in this study was provided by the participants’ legal guardians/next of kin.

## Author contributions

LB: Conceptualization, Formal analysis, Methodology, Writing – original draft. SJ: Conceptualization, Methodology, Writing – review and editing. CS: Conceptualization, Methodology, Writing – review and editing. A-KB: Conceptualization, Methodology, Writing – review and editing. AS: Conceptualization, Methodology, Project administration, Writing – review and editing. UA: Writing – review and editing. SS: Conceptualization, Methodology, Writing – review and editing. RE: Conceptualization, Methodology, Writing – review and editing. TH: Conceptualization, Methodology, Project administration, Supervision, Writing – review and editing.
