# Supplementary material for: Unfavorable nutrient intakes in children up to school entry age: results from the nationwide German KiESEL study
Source: Front Nutr. 2024 Jan 23;10:1302323. doi: 10.3389/fnut.2023.1302323 (PMC10844530; doi:10.3389/fnut.2023.1302323)
Supplement: Supplementary file 1 [file Data_Sheet_1.pdf]

Supplementary Material

Table S1 Health research reporting checklist – based on the STROBE-nut guidelines (1)

		Reporting Item	*Page Number*
**Title and abstract**			
Title	#1a	Indicate the study's design with a commonly used term in the title or the abstract	p. 1
Abstract	#1b	Provide in the abstract an informative and balanced summary of what was done and what was found	p. 1 & 2
None	#nut-1	State the dietary/nutritional assessment method(s) used in the title or in the abstract.	p. 1
**Introduction**			
Background / rationale	#2	Explain the scientific background and rationale for the investigation being reported	p. 2
Objectives	#3	State specific objectives, including any prespecified hypotheses	p. 2
**Methods**			
Study design	#4	Present key elements of study design early in the paper	p. 2 & 3
Setting	#5	Describe the setting, locations, and relevant dates, including periods of recruitment, exposure, follow-up, and data collection	p. 2 & 3
Eligibility	#6a	Cohort study: Give the eligibility criteria and the sources and methods of selection of participants. Describe methods of follow-up. Case-control study: Give the eligibility criteria and the sources and methods of case ascertainment and control selection. Give the rationale for the choice of cases and controls. Cross-sectional study: Give the eligibility criteria, and the sources and methods of selection of participants.	n/a, sampling performed within KiGGS wave 2 (2)
None	#6b	Cohort study: For matched studies, give matching criteria and number of exposed and unexposed. Case-control study: For matched studies, give matching criteria and the number of controls per case.	n/a, cross-sectional study
Variables	#7	Clearly define all outcomes, exposures, predictors, potential confounders, and effect modifiers. Give diagnostic criteria, if applicable	n/a, exploratory dietary survey
Data sources and measurement	#8	For each variable of interest give sources of data and details of methods of assessment (measurement). Describe comparability of assessment methods if there is more than one group. Give information separately for exposed and unexposed groups if applicable.	p. 2 & 3
Bias	#9	Describe any efforts to address potential sources of bias	p. 3
Study size	#10	Explain how the study size was arrived at	n/a, sampling performed within KiGGS wave 2 (2)

		Reporting Item	*Page Number*
Quantitative variables	#11	Explain how quantitative variables were handled in the analyses. If applicable, describe which groupings were chosen, and why	p. 3
Statistical methods	#12a	Describe all statistical methods, including those used to control for confounding	p. 3
Subgroups and interactions	#12b	Describe any methods used to examine subgroups and interactions	p. 3
Missing data	#12c	Explain how missing data were addressed	p. 2
Loss to follow up	#12d	Cohort study: if applicable, explain how loss to follow-up was addressed. Case-control study: if applicable, explain how matching of cases and controls was addressed. Cross-sectional study: if applicable, describe analytical methods taking account of sampling strategy.	n/a, no such methods included
Sensitivity analysis	#12e	Describe any sensitivity analyses	n/a, no sensitivity analysis performed
None	#nut-5	Describe any characteristics of the study settings that might affect the dietary intake or nutritional status of the participants, if applicable.	p. 3
None	#nut-6	Report any particular dietary, physiologic, or nutritional characteristics that were considered when selecting the target population.	n/a, representative sample
None	#nut-7.1	Clearly define foods, food groups, nutrients, or other food components (e.g., preparation method, taxonomical descriptors, classification, chemical form).	p. 3
None	#nut-7.2	When calculating dietary patterns, describe the methods to obtain them and their nutritional properties.	n/a, no dietary patterns calculated
None	#nut-8.1	Describe the dietary assessment method(s) (e.g., portion size estimation, number of days and items recorded, how it was developed and administered, and how quality was ensured); report if and how supplement intake was assessed.	p. 3
None	#nut-8.2	Describe and justify food-composition data used; explain the procedure to match food composition with consumption data; describe the use of conversion factors, if applicable	p. 3
None	#nut-8.3	Describe the nutrient requirements, recommendations, or dietary guidelines and the evaluation approach used to compare intake with the dietary reference values, if applicable	p. 3
None	#nut-8.4	When using nutritional biomarkers, additionally use the STROBE-ME; report the type of biomarkers used and usefulness as dietary exposure markers	n/a, no biomarkers used
None	#nut-8.5	Describe the assessment of nondietary data (e.g., nutritional status and influencing factors) and timing of the assessment of these variables in relation to dietary assessment	p. 3
None	#nut-8.6	Report on the validity of the dietary or nutritional assessment methods and any internal or external validation used in the study, if applicable	p. 9

		Reporting Item	*Page Number*
None	#nut-9	Report how bias in dietary or nutritional assessment was addressed (e.g., misreporting, changes in habits as a result of being measured, data imputation from other sources).	p. 3
None	#nut-11	Explain categorization of dietary/nutritional data (e.g., use of N-tiles and handling of nonconsumers) and the choice of reference category, if applicable.	n/a, no categorization performed
None	#nut-12.1	Describe any statistical method used to combine dietary or nutritional data, if applicable.	n/a, no data combined
None	#nut-12.2	Describe and justify the method for energy adjustments, intake modeling, and use of weighting factors, if applicable	p. 3
None	#nut-12.3	Report any adjustments for measurement error (i.e., from a validity or calibration study).	n/a, no adjustments performed
**Results**			
Participants	#13a	Report numbers of individuals at each stage of study—eg numbers potentially eligible, examined for eligibility, confirmed eligible, included in the study, completing follow-up, and analysed. Give information separately for exposed and unexposed groups if applicable.	p. 2, Table 1–4
Non-participation	#13b	Give reasons for non-participation at each stage	n/a, refer to Nowak et al., 2022 (2)
Participant journey	#13c	Consider the use of a flow diagram	Figure S1
Descriptive data	#14a	Give characteristics of study participants (eg demographic, clinical, social) and information on exposures and potential confounders. Give information separately for exposed and unexposed groups if applicable.	p. 3 & 4, Table 1
Missing data	#14b	Indicate number of participants with missing data for each variable of interest	p. 2 & 4, Table 1, Figure S1
Follow-up time	#14c	Cohort study: Summarise follow-up time (eg, average and total amount)	n/a, cross-sectional study
Outcome data	#15	Cohort study: report numbers of outcome events or summary measures over time. Case-control study: report numbers in each exposure category, or summary measures of exposure. Cross-sectional study: report numbers of outcome events or summary measures.	p. 4 & 7, Tables 1–4, Figure 1 & 2
Main results	#16a	Give unadjusted estimates and, if applicable, confounder-adjusted estimates and their precision (eg, 95% confidence interval). Make clear which confounders were adjusted for and why they were included	Table 3 & 4
Category boundaries	#16b	Report category boundaries when continuous variables were categorized	p. 2 & 3
Relative and absolute risks	#16c	If relevant, consider translating estimates of relative risk into absolute risk for a meaningful time period	n/a, no relative risks included

		Reporting Item	*Page Number*
Other analyses	#17	Report other analyses done—eg analyses of subgroups and interactions, and sensitivity analyses	p. 4 & 7, Table 3 & 4 (toddlers vs. preschoolers)
None	#nut-13	Report the number of individuals excluded on the basis of missing, incomplete, or implausible dietary and nutritional data.	p. 2, Figure S1
None	#nut-14	Give the distribution of participant characteristics across the exposure variables, if applicable; specify if food consumption for the total population or consumers only was used to obtain results	n/a, no exposure variables included
None	#nut-16	Specify if nutrient intakes are reported with or without the inclusion of dietary supplement intake, if applicable.	p. 3, Table 4, Figure 2
None	#nut-17	Report any sensitivity analysis (e.g., exclusion of misreporters or outliers) and data imputation, if applicable	n/a, no sensitivity analysis performed
**Discussion**			
Key results	#18	Summarise key results with reference to study objectives	p. 7
Limitations	#19	Discuss limitations of the study, taking into account sources of potential bias or imprecision. Discuss both direction and magnitude of any potential bias.	p. 9
Interpretation	#20	Give a cautious overall interpretation considering objectives, limitations, multiplicity of analyses, results from similar studies, and other relevant evidence.	p. 7 et seqq.
Generalisability	#21	Discuss the generalisability (external validity) of the study results	p. 9
None	#nut-19	Describe the main limitations of the data sources and assessment methods used and implications for the interpretation of the findings	p. 9
None	#nut-20	Report the nutritional relevance of the findings, given the complexity of diet or nutrition as an exposure.	p. 7 et seqq.
**Other Information**			
Funding	#22	Give the source of funding and the role of the funders for the present study and, if applicable, for the original study on which the present article is based	p. 10
Ethics	#nut-22.1	Describe the procedure for consent and study approval from ethics committee(s).	p. 9
Data statement	#nut-22.2	Provide data collection tools and data as online material or explain how they can be accessed	p. 9

The STROBE-nut checklist is distributed under the terms of the Creative Commons Attribution License CC-BY. This checklist was completed on 09. August 2023 using https://www.goodreports.org/, a tool made by the EQUATOR Network in collaboration with Penelope.ai

Table S2 Calculated cut-off values[1] for the classification of under- and over-reporting of energy intake at individual level[2]

	Age	Under-reporting	Over-reporting
Cut-off values	1–3 years	< 0.92	> 2.12
	≥ 4 years	< 1.06	> 2.42

[1] For the ratio of reported energy intake to estimated basal metabolic rate.
[2] Age specifications refer to completed years of life, e.g., the age group '1–3 years' refers to children aged 1.0 to 3.9 years.

Table S3 EFSA Average Requirements (ARs) for energy (kcal/day) for toddlers and preschoolers (3)

Age	ARs (kcal/day)	
	Boys	Girls
1 year	788[1]	716[1]
2 years	1027[1]	955[1]
3 years	1170[1]	1099[1]
4 years	1266[1]; 1433[2]; 1624[3]	1170[1]; 1337[2]; 1505[3]
5 years	1337[1]; 1529[2]; 1720[3]	1242[1]; 1409[2]; 1600[3]
6 years	1409[1]; 1600[2]; 1815[3]	1314[1]; 1505[2]; 1696[3]

Physical activity level (PAL) = [1]1.4; [2]1.6; [3]1.8

Table S4 EFSA Dietary Reference Values for macro- and micronutrients for toddlers and preschoolers (3)[1]

Nutrients	Type	Dietary Reference Values		
		Toddlers	Preschoolers	
		1–2 years	3 years	4–6 years
**Macronutrients**				
Water (L/day)	AI	1.1–1.2[2]	1.3	1.6
Protein	PRI	1.14[4]	0.9	0.86[7]
Fat (E%)	RI	35–40	35–40	20–35
Carbohydrates (E%)	RI	45–60	45–60	45–60
Fiber (g/day)	AI	10	10	14
**Vitamins**				
Retinol equivalents (µg/day)	PRI	250	250	300
Vitamin D[10] (µg/day)	AI	15	15	15
α-tocopherol (mg/day)	AI	6	9	9
Vitamin K[11] (µg/day)	AI	12	12	20
Thiamin (mg/MJ)	PRI	0.1	0.1	0.1
Riboflavin (mg/day)	PRI	0.6	0.6	0.7
Niacin equivalents (mg/MJ)	PRI	1.6	1.6	1.6
Pantothenic acid (mg/day)	AI	4	4	4
Pyridoxine (mg/day)	PRI	0.6	0.6	0.7
Biotin (µg/day)	AI	20	20	25
Folate equivalents (µg/day)	PRI	120	120	140
Vitamin B12 (µg/day)	AI	1.5	1.5	1.5
Vitamin C (mg/day)	PRI	20	20	30
**Minerals**				
Potassium (mg/day)	AI	800	800	1100
Calcium (mg/day)	PRI	450	450	800
Magnesium (mg/day)	AI	170	230	230
Phosphorus (mg/day)	AI	250	250	440
Iron (mg/day)	PRI	7	7	7
Zinc (mg/day)	PRI	4.3	4.3	5.5
Copper (mg/day)	AI	0.7	1.0	1.0
Manganese (mg/day)	AI	0.5	0.5	1.0
Iodine (µg/day)	AI	90	90	90

[1] AI, Adequate Intake; E%, percentage of energy intake; PRI, Population Reference Intake; RI, Reference Intake Range for Macronutrients.
[2] For children of 1 year of age.
[3] For children of 2 years of age.
[4] For children of 12–17 months of age.
[5] For children of 18–23 months of age.
[6] For children of 24–35 months of age.
[7] For children of 4 years of age.
[8] For children of 5 years of age.
[9] For children of 6 years of age.
[10] Assuming minimal cutaneous vitamin D synthesis.
[11] As phylloquinone.

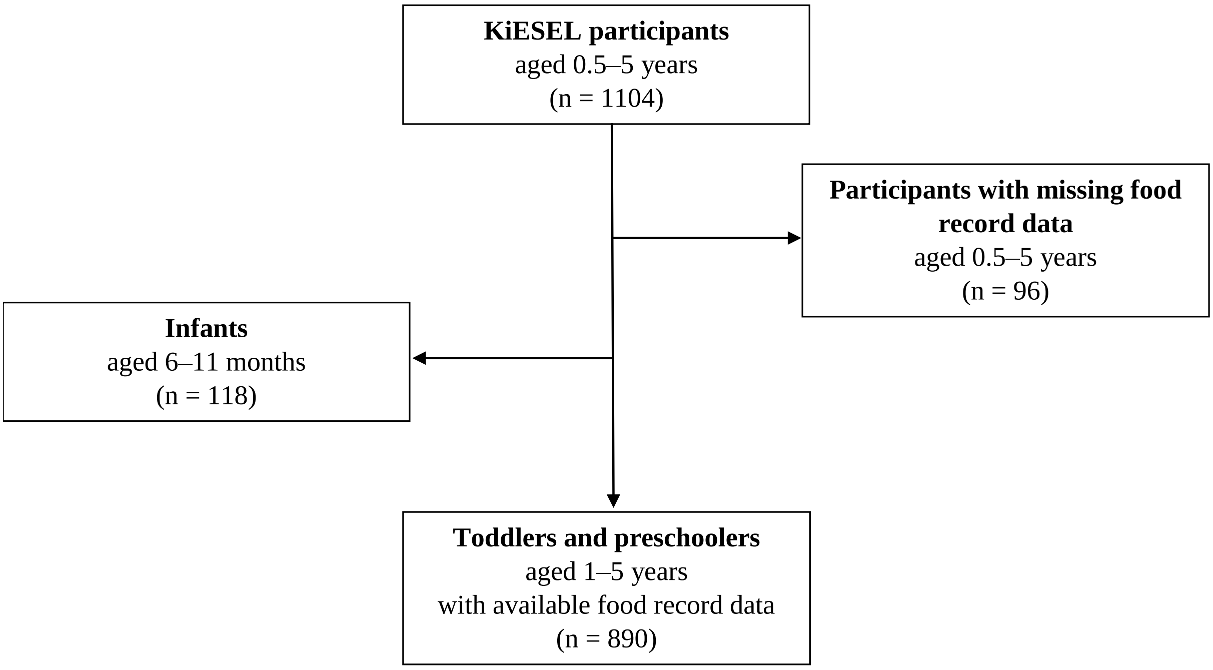

Figure S1 Participant flow chart of the study sample; KiESEL, Children's Nutrition Survey to Record Food Consumption

References

1. Lachat C, Hawwash D, Ocké MC, Berg C, Forsum E, Hörnell A, et al. Strengthening the Reporting of Observational Studies in Epidemiology - Nutritional Epidemiology (Strobe-Nut): An Extension of the Strobe Statement. *Nutr Bull* (2016) 41(3):240–51. Epub 20160814. doi: 10.1111/nbu.12217.
2. Nowak N, Diouf F, Golsong N, Höpfner T, Lindtner O. Kiesel – the Children's Nutrition Survey to Record Food Consumption for the Youngest in Germany. *BMC nutrition* (2022) 8(1):64. doi: 10.1186/s40795-022-00527-6.
3. European Food Safety Authority (EFSA). Dietary Reference Values for Nutrients: Summary Report. *EFSA supporting publications* (2017) 14(12):e15121. doi: 10.2903/sp.efsa.2017.e15121.
